# Evaluating the Effectiveness of Screen-Based Haptic Virtual Reality Simulators in Preclinical Prosthodontic Crown Preparation: Mixed Methods Analysis Study

**DOI:** 10.2196/88916

**Published:** 2026-07-08

**Authors:** Nabil Odisho, Ara Simonian, Rita Chamoun, Maria Christidis, Giancarlo De la Torre Canales, Abhishek Kumar, Anastasios Grigoriadis

**Affiliations:** 1 Department of Dental Medicine Division of Oral Rehabilitation Karolinska Institutet Huddinge, Stockholm Sweden; 2 Department of Neurobiology Care Sciences and Society Karolinska Institutet Huddinge, Stockholm Sweden; 3 Egas Moniz Center for Interdisciplinary Research (CiiEM) Egas Moniz School of Health & Science Caparica, Almada Portugal; 4 Department of Conservative Dentistry and Endodontics, Dr D Y Patil Dental College and Hospital, Dr. D Y Patil Vidyapeeth (Deemed to be University) Pimpri, Pune India; 5 Academic Center for Geriatric Dentistry Stockholm Sweden

**Keywords:** blended learning, crown preparation, dental education, haptic simulation, haptic virtual reality simulation, HVRS, preclinical dentistry, prosthodontics, psychomotor skills, simulation training, total occlusal convergence, virtual reality

## Abstract

**Background:**

Crown preparation is a technically demanding psychomotor skill in undergraduate dental education. While traditional typodont training is the gold standard, it is resource-intensive and difficult to individualize. Screen-based haptic virtual reality simulators (HVRSs) may provide a pedagogical adjunct to conventional training, but their effectiveness in supporting transfer of skills to physical tooth preparation remains unclear.

**Objective:**

This study evaluated whether 3 hours of self-directed HVRS training improved undergraduate dental students’ performance in physical typodont crown preparation compared with no HVRS training. Secondary aims were to examine self-confidence and students’ perceptions of HVRS-based training. Manual dexterity was assessed exploratorily using the Grooved Pegboard Test (GPT).

**Methods:**

A mixed methods study was conducted with 44 fifth-semester dental students at Karolinska Institutet. Participants were allocated to an HVRS training group (n=22) or a control group (n=22). The HVRS group completed 3 hours of self-directed HVRS training over 1 week, whereas the control group received no simulator-based training. Both groups then prepared a maxillary right first molar for a monolithic zirconia crown on a phantom head. Crown preparation quality was assessed using PrepCheck, and a blinded examiner scored 8 areas of interest on a 0-3 grading scale. Manual dexterity was assessed using the GPT. Self-confidence was evaluated in both groups using survey items, while perceptions of the HVRS were evaluated only among HVRS group participants. Free-text responses from the HVRS group were analyzed using inductive thematic analysis.

**Results:**

The HVRS group achieved a higher mean total preparation score than the control group, but the difference was not statistically significant (11.9 vs 10.9; *P*=.24). In unadjusted analyses, the HVRS group scored higher for total occlusal convergence (*P*=.04), but this difference did not remain statistically significant after Bonferroni correction for 8 area-of-interest comparisons. Manual dexterity measured by the GPT improved in both groups, but the control group was significantly faster at baseline (*P*=.04) and postintervention (*P*=.001). Self-confidence ratings were broadly similar between groups; very low confidence was reported by 5% (1/20) of respondents in the HVRS group and 18% (4/22) in the control group. Most HVRS group respondents rated the HVRS drilling sensation as having limited comparability with typodont teeth and natural teeth. Qualitative responses suggested that students valued the HVRS for understanding procedural steps, applying theoretical knowledge, and allowing repeated practice, while reported challenges included limited realism, visual-tactile disconnect, and occasional technical issues.

**Conclusions:**

Three hours of self-directed HVRS training did not significantly enhance overall crown preparation quality on typodont teeth or improve students’ general self-confidence. There is preliminary indication that HVRS could assist in mastering specific geometric parameters like total occlusal convergence. Future randomized controlled trials with stratified baseline dexterity and larger sample sizes are required to determine the optimal role of HVRS in dental education.

## Introduction

### Overview

Undergraduate dental education has traditionally followed a curriculum divided into 2 main components: theoretical knowledge and clinical skills [1]. Given the irreversible nature of most operative procedures in dentistry, students must acquire well-developed psychomotor skills to ensure the safe and effective delivery of patient treatment and care [2-4]. Traditionally, phantom heads equipped with typodont models have been used in preclinical courses to teach students technical skills and manual dexterity [5-8]. These simulators remain widely used, particularly to teach tooth preparation for crowns, which is among the most challenging tasks for students to master. Proficiency in such procedures requires both psychomotor skills and knowledge of tooth anatomy and tissue structures to achieve the characteristics needed for a clinically successful crown [9].

### Traditional Preclinical Training and Its Limitations

Training with typodont models has been shown to be both effective and cost-efficient in enhancing the psychomotor skills of dental students [2,5,10]. However, this method presents several limitations [2,11]. In many preclinical sessions, feedback is largely end point oriented: students typically work independently until the preparation is perceived as “nearly finished,” and then request faculty feedback [10,12-14]. Faculty, therefore, evaluate the final preparation and suggest corrective adjustments, but may not observe the procedural steps that produced the outcome. This can allow process misunderstandings to persist, for example, incorrect bur positioning and angulation. Moreover, it is challenging to individualize training due to the absence of realistic internal anatomy, such as enamel, dentin, and pulp chambers, in many standard plastic models. Some universities have used human extracted teeth for preclinical training. However, their use is increasingly questioned due to ethical and legal dilemmas regarding donor consent and whether extracted tissues remain the property of the patient [15]. Natural teeth could also pose biosafety risks because they are potential sources of blood-borne pathogens that can be transmitted through aerosols or sharp injuries during laboratory practice [15]. Environmental concerns have also been raised regarding the amount of plastic waste generated by acrylic teeth and their impact on the climate. Additionally, the equipment in the preclinic requires regular maintenance, handpieces require replacement, and water drainage and suction systems are necessary, all of which contribute to additional costs.

### Haptic Virtual Reality Simulation in Dental Education

To address the challenges of traditional approaches, particularly limited realism in terms of tactile fidelity (tissue resistance) and visual fidelity, various methodologies have been developed [10,13]. One such methodology is the use of virtual reality (VR) simulators, which were first implemented in dental schools in the early 2000s [2]. Within dental education, VR is used as an umbrella term to describe technologies ranging from full 3D headsets to systems that perform automated assessment of students [16]. To increase the realism of these simulators, haptic technology was later integrated, leading to the development of screen-based haptic virtual reality simulators (HVRSs) [2,17]. These systems provide users with tactile force feedback through a handpiece while they view a 3D screen [18]. The force feedback simulates the physical resistance of different virtual dental tissues. These HVRSs offer several advantages, including the ability to be improved and individualized more easily, as well as facilitating objective assessment of students’ psychomotor skills [18,19]. However, certain limitations persist, such as high purchase costs, long implementation times, and the fact that many systems have not yet been validated.

Several studies have explored the integration of HVRSs into conventional dental education and suggest that these systems may be useful adjuncts for developing manual dexterity and motor skills [20-25]. However, studies evaluating the transfer of skills acquired in virtual environments to physical clinical or typodont-based performance have reported mixed findings [21,24-26]. Most previous HVRS research has focused on operative dentistry and endodontic procedures [24,25], whereas prosthodontic procedures, particularly crown preparation, have received less attention. This is important because crown preparation is technically demanding and requires precise control within a restricted working field [9].

Although HVRSs are becoming more common in dental education, there are still questions about how these systems should be used in undergraduate training. Previous studies have evaluated HVRSs under different educational conditions, with variations in training duration, curricular integration, and the extent of feedback or instructional support [21,24-26]. However, many of these protocols involve greater simulator exposure than is typically feasible in routine dental education. Given the time constraints of undergraduate curricula, it is important to determine whether shorter periods of self-directed HVRS training can improve students’ performance before they undertake their first physical crown preparation exercise.

Therefore, the aim of this study was to evaluate whether brief HVRS training, delivered as 3 hours of self-directed practice over 1 week, improved students’ performance in physical typodont crown preparation compared with a control group that did not receive HVRS training or any other simulator training. Secondary aims were to examine self-reported self-confidence and HVRS group participants’ perceptions of HVRS-based training. Manual dexterity was assessed exploratorily using the Grooved Pegboard Test (GPT). We hypothesized that students who received HVRS training would achieve higher total crown preparation scores and report higher self-confidence than students in the control group.

## Methods

### Ethical Considerations

The study was conducted in accordance with the Declaration of Helsinki and was approved by the Ethics Review Authority, Stockholm (Dnr 2023-04136-01). Written informed consent was obtained from all participants, ensuring voluntary participation. Participants were also informed that they were free to withdraw from the study at any time, for any reason, without needing to provide an explanation.

### Participant Selection

All 83 dental students in the fifth semester at Karolinska Institutet were invited to participate in the study. A total of 48 students initially agreed to participate. However, 4 students later withdrew due to scheduling conflicts. The required sample size was estimated using G*Power (version 3.1.9.7). An a priori power analysis was performed assuming an effect size of *f*=0.15, a significance level (α) of .05, and statistical power (1 − β) of .80. The study design included 2 groups with 8 repeated measurements. Based on these parameters, the analysis indicated that a minimum total sample size of 42 participants was required to detect the expected interaction effect. The analysis yielded an actual statistical power of 0.813. Accordingly, 44 students were finally recruited for the study and divided into an HVRS group and a control group. Participants were allocated using a nonrandomized procedure based on prior self-reported HVRS exposure and schedule availability. Specifically, participants were asked, “Have you practiced on SIMtoCARE in the past six months?” If they responded “yes,” they were enrolled in the HVRS group; if not, they were allocated according to their study schedule and availability to either of the 2 groups. It should also be noted that, at the time of the study, none of the students had yet begun any crown preparation in their preclinical prosthodontics course.

During their previous restorative dentistry preclinical course in the fourth semester, all students were introduced to the HVRS and had the opportunity to practice independently outside scheduled teaching. HVRS practice was not mandatory in the fourth semester. Because the study started at the beginning of the fifth semester in September, prior HVRS exposure was assessed only for the 6 months immediately preceding the start of the study. Self-reported prior use ranged from 1 to 4 hours for 7 students, while 3 students reported approximately 4 to 8 hours of use. The demographic characteristics and baseline prior simulator exposure of both groups are summarized in Table 1.

**Table 1:** Participant demographics, baseline characteristics, and prior haptic virtual reality simulator (HVRS) exposure.

Characteristics		HVRS group (n=22)	Control group (n=22)
Age (years), mean (SD); range		24.1 (4.6); 20-40	22.7 (1.6); 20-27
**Sex, n (%)**			
	Female	14 (64)	14 (64)
	Male	8 (36)	8 (36)
Prior HVRS use^a^ within the previous 6 months, n (%)		10 (45)	0 (0)
**Self-reported prior HVRS use among exposed students (hours), n**			
	1-4	7	—^b^
	4-8	3	—

^a^Prior HVRS use was self-reported; duration estimates were approximate.

^b^Not applicable.

### Intervention

**Figure 1:**
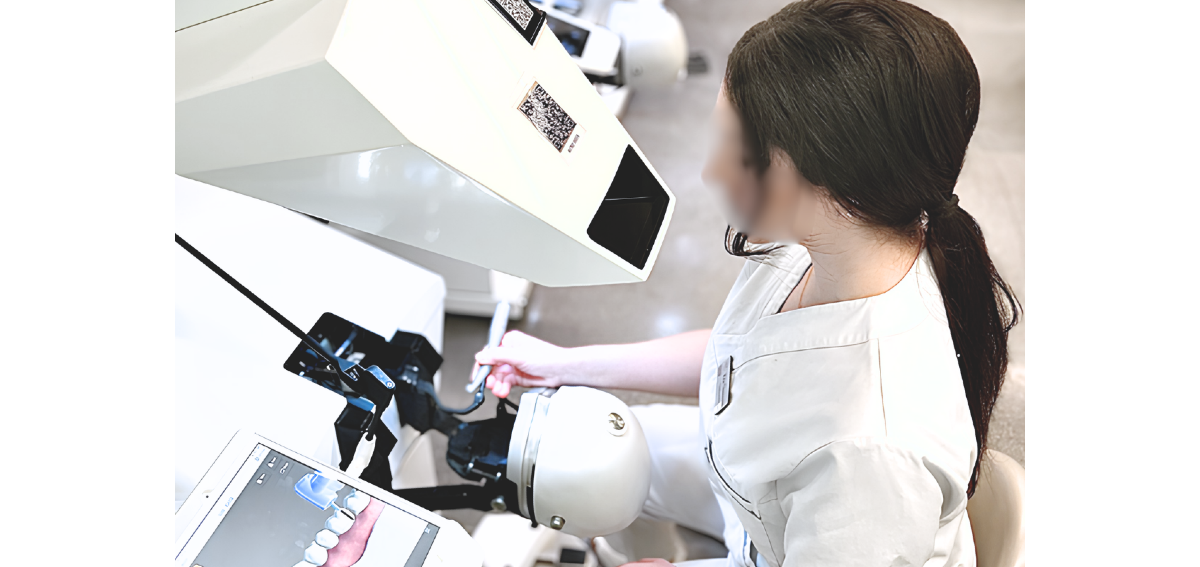
SIMtoCARE Dente screen-based haptic virtual reality simulator used in the study. The system includes a physical handpiece connected to a force-feedback arm and a 3D display–based visual interface.

The HVRS group viewed a 10-minute recorded demonstration showing the tooth preparation of the maxillary right first molar for a monolithic zirconia crown. After the demonstration, participants received access to the SIMtoCARE Dente HVRS (SIMtoCARE), which is a screen-based haptic virtual reality simulator (Figure 1). The system allows the user to manipulate a physical handpiece connected to a force-feedback arm while viewing the operative field on a 3D display. The system also includes a dental mirror handle and a foot pedal for handpiece speed control. In addition, the platform includes courseware with manual dexterity exercises and dental procedures of varying complexity across several dental disciplines.

Each student was assigned a unique identification number and login credentials. A dedicated course was created within the Dente software specifically for this study, containing 2 cases: “MAN-05” and “04-Crown.” Participants in the HVRS group completed a total of 3 hours of HVRS training over the course of 1 week. The training consisted of 2 sessions, each lasting 90 minutes, with 10-minute rest periods during the sessions.

The training began with a live demonstration of how to use the HVRS, followed by the manual dexterity task “MAN-05” to familiarize participants with the system and help them understand how to operate the HVRS. Participants were instructed to aim for predetermined performance targets (minimum 95% target accuracy, maximum 10% leeway, and 0% container score) as an optimal goal during this initial task. To avoid reducing the time available for the crown preparation exercise, participants practiced “MAN-05” for up to 10 minutes; if the target level was not achieved within this time, they nevertheless proceeded to the crown preparation task. After completing the manual dexterity task, students practiced preparing the maxillary right first molar for a monolithic zirconia crown. No faculty-provided instructional feedback was given during training, apart from assistance with technical issues. Students also had access to a physical, 3D-printed demonstration model of what an ideal preparation should look like. This model was also available during the preparation examination for all participants. The HVRS interface displayed quantitative percentage-based performance indicators (eg, target removal, leeway removal, and container removal) to guide task completion for the manual dexterity case, but it did not provide individualized feedback beyond these metrics.. Therefore, the HVRS training was mainly intended to help students become familiar with the procedure by watching the demonstration, practicing repeatedly, comparing their work with the ideal model, and using the basic indicators provided by the system.

The control group did not receive any HVRS or other simulator-based training. They only viewed the same 10-minute demonstration video immediately before completing the preparation examination. Within 1 week after completing the training period, both groups undertook the crown preparation examination. Students prepared the maxillary right first molar for a monolithic zirconia crown using the Frasaco AG-3 model mounted on a phantom head mannequin. Participants were assigned 90 minutes to complete the examination, which was designed to be similar to the end-of-course preparatory examination in the preclinical prosthodontics course, with the only difference being that participants were provided with an additional 15 minutes of preparation time than the standard examination.

### Outcomes

The primary outcome was overall crown preparation quality, measured as the total examiner evaluation score, calculated as the sum of 8 areas of interest (AOIs). Secondary outcomes were the individual AOI subscores and participant-reported outcomes from the survey, including self-confidence in both groups and perceptions of the HVRS among HVRS group participants. Manual dexterity was assessed exploratorily using the GPT.

### Dexterity Assessment

The GPT was chosen as an exploratory baseline and tracking measure of general manual dexterity in both groups [27]. This test requires participants to insert pegs into slots with randomly positioned holes using only 1 hand. The GPT is widely used in neuropsychological examinations to evaluate fine motor function and has been associated with central nervous system function. It is the most frequently used fine motor function test in the United States, Canada, and Nordic countries [27,28].

The GPT was administered to the HVRS group both before and after HVRS training, but to the control group before and after the preparation examination. The groups were tested at different time points to measure the immediate psychomotor impact of their respective primary physical tasks. For the HVRS group, the GPT was administered immediately before and after the HVRS sessions to evaluate the direct effect of the haptic intervention. Because the control group did not participate in HVRS training, the GPT was administered immediately before and after the physical crown preparation examination. To standardize testing conditions, a 10-second waiting period was implemented at the start of each trial: the timer was activated, and participants waited for 10 seconds before beginning the task. This ensured that all participants started under the same conditions. The initial 10-second delay was subsequently subtracted from the total recorded time.

### Digital Assessment Using PrepCheck

All assessments were performed by a faculty member from the Department of Dental Medicine, Division of Oral Rehabilitation at Karolinska Institutet. To ensure objectivity, evaluations were conducted in a blinded manner using only student identification numbers. Crown preparations were evaluated using a digital approach similar to that used in the validation studies by Schepke et al [29]. Each crown preparation was first scanned by the examiner using a CEREC Omnicam intraoral scanner (Dentsply Sirona) with CEREC SW 22 and analyzed with PrepCheck (version 5.0). The prepared tooth was then replaced with an unprepared standard plastic tooth of the same type (maxillary right first molar) and scanned again as a reference for evaluating occlusal and axial reduction. The margin line for each prepared crown was manually drawn before proceeding with the PrepCheck application. The software generated quantitative parameters that were visualized through a color-coded system and expressed as percentage deviations from ideal values.

To evaluate the preparations, a customized grading scale based on the Baylor College of Dentistry model was adopted to meet the educational standards at Karolinska Institutet [30]. The examiner evaluated 8 key AOIs for each crown preparation: occlusal reduction, axial reduction, total occlusal convergence (TOC; taper angle between opposing axial walls), preparation walls and surfaces, undercuts, margin line design, finishing of the margin line, and damage to adjacent teeth. The grading scale ranged from 0 to 3, with 0 indicating significant errors that could compromise the final restoration, 1 indicating major errors, 2 indicating minor deviations from the ideal, and 3 indicating optimal preparations suitable for clinical success.

To ensure objectivity and directly address the outputs of the digital assessment, the specific thresholds and visualizations generated by PrepCheck were integrated into the customized grading scale where applicable. TOC was the only AOI scored solely from a single numerical angular output generated by PrepCheck because this parameter provides a quantitative measurement of preparation taper. For the remaining AOIs, including occlusal reduction, axial reduction, undercuts, margin design, finish line quality, and preparation walls and surfaces, the examiner used PrepCheck measurements and visualizations together with clinical interpretation to assign scores on the 0-3 rubric. Iatrogenic damage to adjacent teeth was assessed visually by the examiner without reliance on the digital software. The complete grading scale, including quantitative thresholds where applicable and clinical evaluation criteria for all assessed parameters, is provided in Multimedia Appendix 1.

### Experience and Self-Confidence Evaluation

Self-confidence was assessed with a single item: “To what extent did you have confidence in your own ability to prepare tooth 16 on typodont models?” Responses were rated on a 4-point verbal scale ranging from “to a very small extent” to “to a very large extent.” For reporting, these categories were summarized as very low, quite low, quite high, and very high self-confidence. In addition, the HVRS group completed three perception items rating (1) comparability of HVRS preparations to typodont preparations, (2) comparability to natural teeth, and (3) perceived usefulness for self-directed training of manual skills, using the same response options. Responses were treated as ordinal categories and summarized as counts and percentages. Additional perception items assessed user-friendliness, likelihood of recommending the HVRS, and intention to use it for additional manual skills using parallel 4-point verbal response scales. The questionnaire also included open-ended questions about participants’ experiences with the HVRS. The qualitative analysis was based on free-text responses to 2 open-ended questions: “What aspects of the simulator did you find useful or beneficial?” and “What aspects of the simulator did you find less useful or less satisfactory?” These responses were used to identify perceived benefits and limitations of the HVRS. The full questionnaire is provided in Multimedia Appendix 2.

The free-text responses regarding the perceived advantages and disadvantages were analyzed qualitatively using inductive thematic analysis [31]. One researcher performed the analysis. First, responses were read repeatedly to achieve familiarity with the data. Next, initial codes were generated across the dataset and then collated into candidate themes. Themes were iteratively reviewed against the coded data and the full dataset and then defined and named to capture the central patterns in participants’ responses. Because the analysis was conducted by a single coder, intercoder reliability was not assessed.

### Statistical Analysis

Statistical analyses were performed using SPSS Statistics (version 27; IBM Corp). Data distribution was assessed using the Shapiro-Wilk test and visual inspection of histograms and quantile-quantile plots. For continuous variables, group comparisons were performed using the independent samples *t* test (2-tailed) for normally distributed data and the Mann-Whitney *U* test for nonnormally distributed data. Associations between categorical variables, including study group and self-confidence, were evaluated using the chi-square test. A 2-tailed *P* value <.05 was considered statistically significant. To control for family-wise error due to multiple AOI comparisons, a Bonferroni-corrected significance threshold was applied for the 8 AOI comparisons (*P*<.006). In addition to statistical significance testing, appropriate effect sizes were calculated and reported to facilitate interpretation of the magnitude of observed effects.

For the exploratory direction-specific PrepCheck TOC analysis, mean values and SDs in degrees were reported for each group. Between-group comparisons were performed using the Mann-Whitney *U* test. This analysis was considered exploratory and was not used as the primary basis for statistical inference.

## Results

### Overview

**Figure 2:**
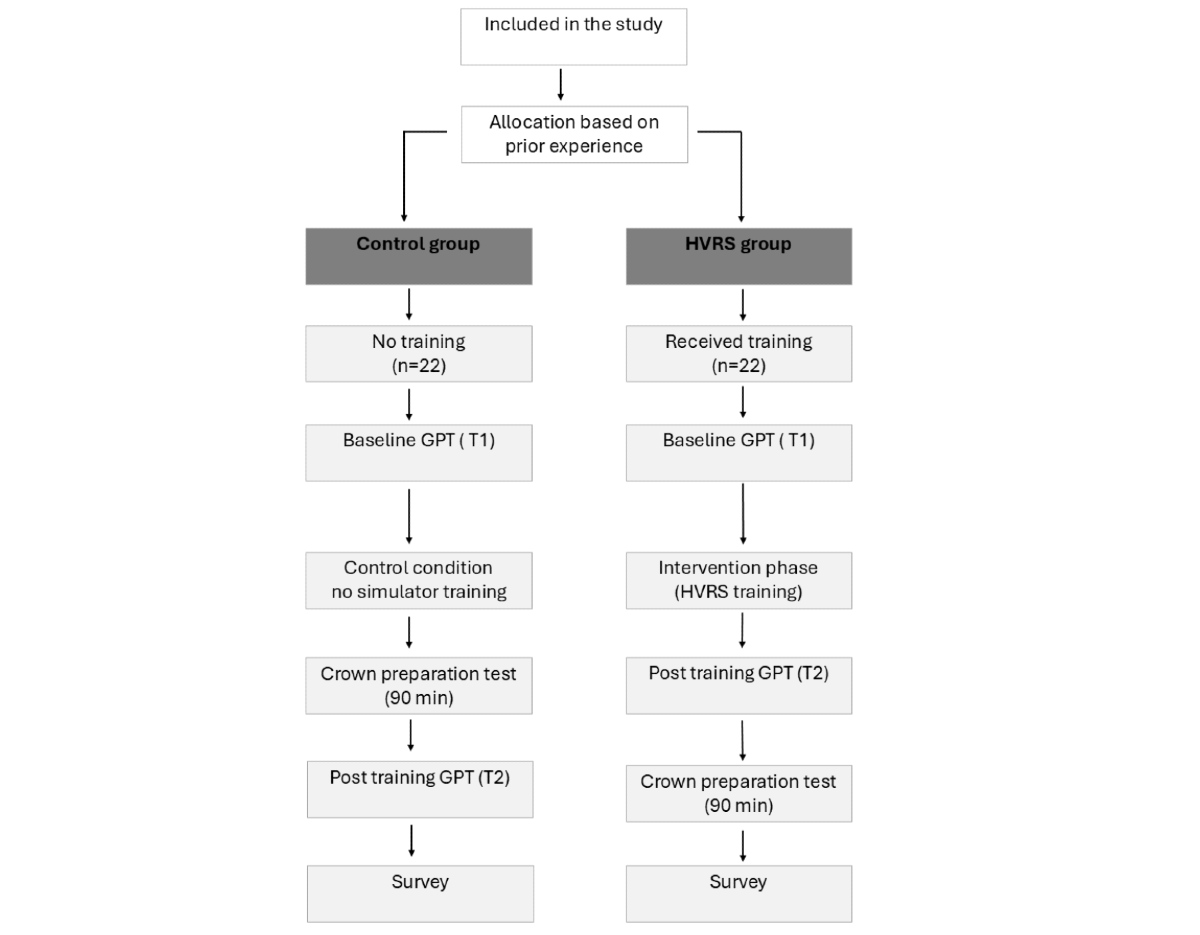
Schematic diagram of the study flow. Phases of allocation, intervention, and assessment are shown for the haptic virtual reality simulator (HVRS) and control groups. GPT: Grooved Pegboard Test.

All 44 participants from both groups were able to complete the entire experimental session reliably (Figure 2).

### Crown Preparation Quality

**Figure 3:**
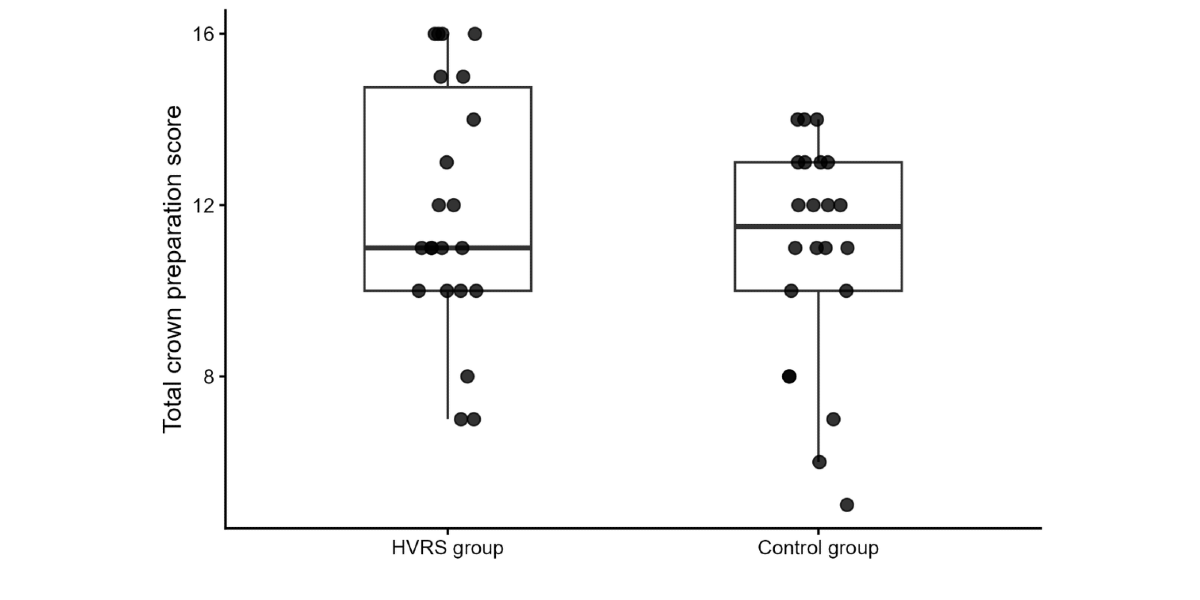
Distribution of total crown preparation scores in the haptic virtual reality simulator (HVRS) and control groups. Individual data points are shown with boxplots. Scores range from 0 to 24, with higher scores indicating better crown preparation quality.

Crown preparation quality was assessed by a blinded examiner. The mean total evaluation score was 11.9 (SD 2.9) for the HVRS group and 10.9 (SD 2.6) for the control group. The distribution of individual total crown preparation scores is shown in Figure 3. This difference in total scores was not statistically significant (*P=.*24). The analysis of the individual AOIs showed a significant difference in one domain (Table 2). Preliminary analysis indicated that the HVRS group received significantly better scores in achieving the correct TOC compared with the control group (*P=*.04). The TOC reflects the taper of the crown preparation and describes the angle formed between opposing axial walls; an appropriate taper is essential for adequate retention and resistance form. However, after adjustment for multiple comparisons across the 8 AOIs using the Bonferroni correction (adjusted significance level α=.006), this difference was no longer statistically significant. The observed effect size was small (*f*=−0.30), indicating a modest magnitude of the group difference. No statistically significant between-group differences were observed for the other AOIs, including occlusal reduction, axial reduction, preparation walls and surfaces, undercuts, margin design and placement, finish line, and neighboring tooth injuries (Table 2).

**Table 2:** Examiner-assigned crown preparation scores by area of interest (AOI). Scores range from 0 to 3, with higher scores indicating better preparation quality.

AOI	HVRS group (n=22), mean (SD)	Control group (n=22), mean (SD)	Unadjusted *P* valueᵃ
Occlusal reduction	1.32 (0.65)	1.23 (0.53)	.66
Total occlusal convergence	1.14 (0.83)	0.64 (0.73)	.04
Axial reduction	1.45 (0.67)	1.27 (0.63)	.42
Walls and surfaces of the preparation	1.64 (0.49)	1.64 (0.66)	.83
Undercuts	2.45 (0.8)	2.59 (0.59)	.74
Margin design and placement	1.14 (0.64)	0.86 (0.56)	.14
Finish line	1.55 (0.67)	1.23 (0.75)	.13
Neighboring tooth injuries	1.32 (0.78)	1.45 (0.51)	.53

^a^*P* values are unadjusted 2-tailed between-group comparisons calculated using the Mann-Whitney *U* test. Because 8 AOI comparisons were performed, the Bonferroni-corrected significance threshold was *P*<.006.

An exploratory direction-specific analysis of PrepCheck-derived TOC values was also performed. In the buccopalatal direction, the mean TOC was 22.22° (SD 6.24°) in the HVRS group and 25.75° (SD 9.79°) in the control group, with no statistically significant between-group difference (*P*=.14). In the mesiodistal direction, the mean TOC was 22.15° (SD 8.85°) in the HVRS group and 29.10° (SD 9.42°) in the control group, and an unadjusted between-group difference was observed (*P*=.03; Multimedia Appendix 3).

### Manual Dexterity

**Figure 4:**
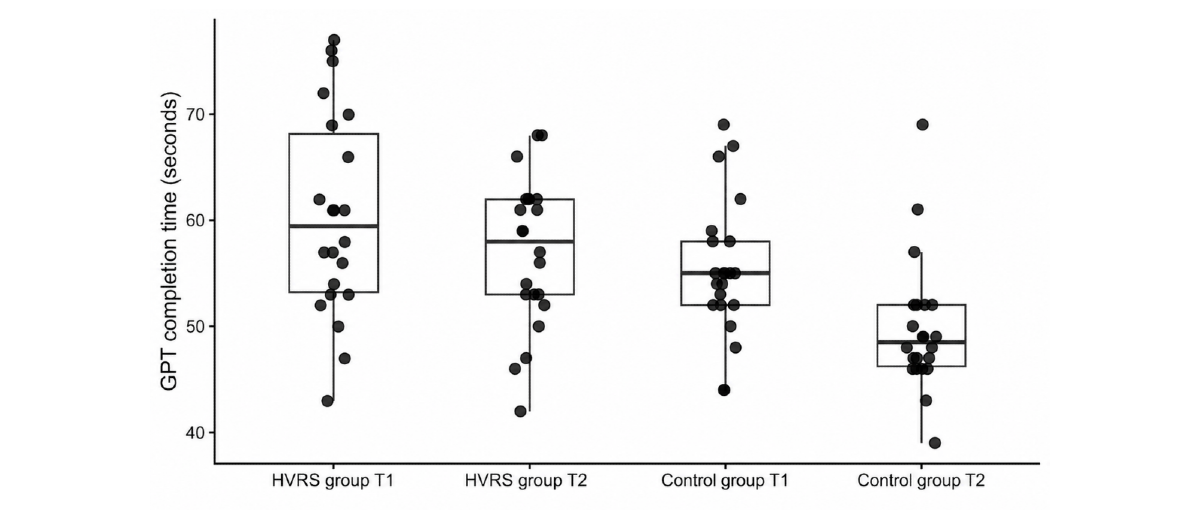
Distribution of Grooved Pegboard Test (GPT) completion times at baseline (T1) and postintervention (T2) in the haptic virtual reality simulator (HVRS) and control groups. Individual data points are shown with boxplots. Lower completion times indicate better manual dexterity performance.

Manual dexterity performance was assessed using the GPT at baseline and postintervention, with individual completion times shown in Figure 4. A significant between-group difference in completion time was present at baseline, with the control group completing the GPT faster than the HVRS group (*P*=.04). Both groups demonstrated shorter completion times at the second assessment. The mean reduction from baseline to postintervention was 5.5 seconds for the control group and 3.5 seconds for the HVRS group. The between-group difference in completion time remained statistically significant at the postintervention assessment (*P*=.001).

### Participant-Reported Outcomes

#### Self-Confidence

A total of 20 out of 22 participants in the HVRS group responded to the self-confidence item. In the HVRS group, 5% (1/20) of participants reported “very low,” 50% (10/20) reported “quite low,” 35% (7/20) reported “quite high,” and 10% (2/20) reported “very high” self-confidence in performing a crown preparation on a typodont tooth. In the control group, 18% (4/22) reported “very low,” 45% (10/22) reported “quite low,” 32% (7/22) reported “quite high,” and 5% (1/22) reported “very high” self-confidence. There was no significant association between group and self-confidence level (*P*=.56).

#### HVRS Perceptions

A total of 20 out of 22 participants in the HVRS group completed the perception items. Regarding perceived realism, for comparability of the HVRS’s drilling sensation to drilling a typodont tooth, 70% (14/20) of participants rated comparability as “quite low,” 15% (3/20) as “very low,” and 15% (3/20) as “quite high.” For comparability to drilling a natural tooth, 45% (9/20) of participants rated comparability as “quite low,” 30% (6/20) as “very low,” and 5% (1/20) as “quite high”; 20% (4/20) indicated they had not yet drilled a natural tooth.

Regarding usability and perceived value, 75% (15/20) of participants rated the HVRS’s user-friendliness (functions and settings) as “quite high,” 5% (1/20) as “very high,” and 20% (4/20) as “quite low.” For usefulness for self-directed training of manual skills, 50% (10/20) of participants rated usefulness as “quite high,” 35% (7/20) as “quite low,” and 15% (3/20) as “very low.” For the likelihood of recommending the HVRS to a peer, 35% (7/20) of participants responded “quite high,” 10% (2/20) responded “very high,” and 55% (11/20) responded “quite low.” For intention to use the HVRS to practice additional manual skills, 25% (5/20) of participants responded “quite high,” 20% (4/20) responded “very high,” and 55% (11/20) responded “quite low.”

### Qualitative Analysis of the Survey

#### Overview

A total of 20 out of the 22 participants in the HVRS group responded to the survey. Below is a qualitative analysis of their responses to the free-text questions about what advantages and disadvantages they perceived the HVRS to have, organized by themes.

#### Advantages of the HVRS

##### Understanding Workflows and Principles

Many participants answered that practicing with the HVRS provided a step-by-step preparation overview of the preparation process.

It was a good way to practice the method itself, for example, starting with separation, then preparing the buccal side, then the palatal side.

It gives a comprehensive overview of the entire process and helps understand what is required when working with a patient.

##### Application of Theoretical Knowledge

Participants mentioned that HVRS helped them apply theoretical knowledge in a practical setting, which improved their understanding of theoretical preparation principles:

You get to practice applying the theory behind the process.

You understand the principles of prepping, like thinking about the preparation margins.

##### Repetitive Practice and Error Correction

Participants also mentioned the advantages of being able to reset and repeat the procedures, which allowed the students to improve techniques and correct mistakes:

It's good that you can reset and start over if something goes wrong.

It is good that you are able to train repeatedly in a way that's not possible with plastic teeth, because you only have to press on “reset.”

#### Disadvantages of the HVRS

##### Lack of Realism

One of the disadvantages that was cited by many of the participants was that the HVRS system lacked tactile and visual realism compared to real teeth or plastic teeth: “It doesn’t feel real” and “It doesn’t feel like preparation in plastic or real teeth.”

##### Tactile and Visual Disconnect

It was challenging for some of the participants to synchronize what they saw with the tactile feedback they received from the HVRS.

The connection between vision and hand sensation somehow feels strange, and you have no perception of where you are.

You don’t get the same feeling when using the drill/air turbine handpiece and to have Coast speed.

##### Technical Limitations and Lag

Some participants reported occasional lagging of the HVRS system as an issue: “It also lags occasionally, which is annoying” and “You can get stuck with the drill, leading to grooves.”

## Discussion

### Overview

This study aimed to evaluate whether 3 hours of HVRS training improved crown preparation performance on typodont teeth compared with a control group. Additionally, the study examined students’ self-confidence and perceptions regarding HVRS-based training, while manual dexterity was assessed exploratorily using the GPT.

### Main Findings

The primary finding was that 3 hours of self-directed HVRS training did not significantly improve overall crown preparation performance compared with the control group (mean total score: 11.9, SD 2.9 vs 10.9, SD 2.6; *P*=.24). Unadjusted analysis indicated that the HVRS group achieved better scores in the TOC (*P*=.04). However, this difference did not remain statistically significant after Bonferroni correction across the 8 AOIs. The direction-specific PrepCheck analysis showed similar results for the HVRS group, particularly in the mesiodistal direction, but this analysis was exploratory and unadjusted for multiple testing. Taken together, these findings suggest that TOC may be a parameter of interest for future studies.

Furthermore, while manual dexterity improved in both groups, the control group remained faster on the GPT at both baseline and postintervention (*P*=.04 and *P*=.001, respectively). Finally, participant-reported outcomes suggested broadly comparable self-confidence across groups, and while perceptions of tactile realism were generally low, students perceived value in the HVRS for understanding procedural workflows.

### Comparison With Prior Work

The absence of a statistically significant difference in total crown preparation performance is consistent with the study by Hattori et al [32] showing that HVRS training does not necessarily lead to broad improvements across all preparation outcomes. Crown preparation is a complex procedure that requires simultaneous control of occlusal reduction, axial reduction, taper, margin design and placement, and avoidance of adjacent tooth damage. A short period of self-directed HVRS training may therefore be insufficient to improve overall performance across all domains of a physical crown preparation task. Previous studies have reported more positive effects of HVRS training for less complex operative procedures. For example, Farag et al [33] reported improvements in several cavity-preparation features after HVRS training. San Diego et al [34] found that students trained with haptic simulation did not perform as well as students trained with conventional approaches in a more demanding caries-removal task. Although the task and study design differ from this study, these findings support the view that HVRS may not produce uniform benefits across all operative or prosthodontic procedures. The educational value of HVRS may therefore depend on how closely the simulator task aligns with the assessed physical task, the duration of training, and the extent of instructional support.

Recent randomized evidence also indicates that VR-based simulator training may have educational value even when it does not outperform conventional simulation. In a randomized controlled trial of veneer tooth preparation, Li et al [35] found no statistically significant differences in training outcomes between VR simulator training and traditional phantom-head training. Such findings should not be interpreted as evidence of equivalence unless formal equivalence testing has been performed. However, they suggest that comparable observed outcomes may still be educationally meaningful when simulator-based training provides additional opportunities for repeated, self-directed practice.

The participant-reported perceptions in this study are consistent with previous reports of limited perceived tactile realism in HVRS. Most HVRS group participants rated the drilling sensation as having limited comparability with typodont teeth or natural teeth. Similar findings have been reported by San Diego et al [34], who found that traditional phantom-head simulators were perceived as more realistic, and by Philip et al [36] and Zafar et al [37], who reported that students often perceived hardness, texture, and tactile sensation in HVRS as insufficiently realistic. These findings suggest that limited tactile and visual fidelity remains a recurring challenge in HVRS-based dental education.

However, limited tactile realism does not necessarily mean that HVRS training has no educational value. De Boer et al [23] demonstrated that psychomotor skills acquired under one level of force feedback could be transferred to another level of force feedback within a virtual learning environment. This suggests that variation in haptic feedback may not, by itself, prevent learning transfer. Nevertheless, because this study did not directly assess transfer between different force-feedback conditions, this interpretation should be regarded as explanatory rather than confirmatory.

Beyond perceived realism and technical fidelity, the qualitative findings provide further insight into how students experienced the educational value of HVRS training. The thematic analysis conducted in this study revealed that students value HVRS for several reasons, including an improved understanding of procedural steps and the ability to practice repeatedly through simple case resets. These findings are consistent with a similar thematic analysis by Ba-Hattab et al [25], which identified “opportunities for repeated practice” and “improved understanding of dental structures” as primary benefits of HVRS. The opportunity for repeated practice may support procedural familiarization and allow students to refine their approach in a low-risk environment.

In addition to performance outcomes, the participant-reported confidence data provide a perspective on how students experienced the preparation task. Although overall self-confidence did not differ significantly between groups, fewer students in the HVRS group reported “very low” confidence compared with the control group. This pattern should not be interpreted as evidence of an intervention effect, but it may suggest that brief HVRS exposure could reduce perceived unpreparedness for some students.

Based on the present findings and prior literature, HVRS may be more appropriately investigated as an adjunctive component within a blended learning model rather than as a replacement for conventional phantom-head training [20,25,33]. HVRS can serve as an introductory tool to familiarize students with procedural steps in crown preparation, after which they transition to phantom heads to develop tactile feel and adapt to the physical environment.

### Methodological Considerations

An important methodological consideration in this study is that the control group demonstrated superior baseline manual dexterity, as measured by the GPT. This preexisting difference in dexterity complicates a straightforward comparison of the 2 groups, as part of the HVRS group’s performance may reflect lower initial psychomotor capacity rather than the effect of HVRS training alone. In addition, the HVRS group included 10 of 22 students who had practiced with the HVRS during the 6 months before the study, whereas none of these students were allocated to the control group.

Because prior HVRS use was optional and allocation was nonrandomized, these imbalances make it difficult to determine whether the observed differences reflect the intervention, baseline psychomotor differences, prior HVRS exposure, self-selection, or a combination of these factors. It is possible that the HVRS group included students who sought additional practice because they perceived a greater need to improve their manual skills. Conversely, these students may have been more motivated and more actively engaged in the curriculum, making them more likely to participate in optional HVRS practice.

### Strengths and Limitations

The study’s strengths include the use of objective digital assessments, such as PrepCheck, and the inclusion of both quantitative and qualitative data to capture students’ performance and perceptions. Nevertheless, several limitations should be acknowledged. The small sample size may have reduced the statistical power to detect subtle differences between groups. One of the limitations is the nonrandomized allocation of participants. Because students with prior HVRS exposure were assigned to the HVRS group to maintain a control group without recent HVRS exposure, selection bias may have been introduced, and the effect of the intervention may have been confounded by prior experience. In addition, the baseline imbalance in manual dexterity between groups limits the extent to which differences in performance can be attributed to the HVRS intervention alone.

The assessment relied on a single blinded examiner, which introduces the possibility of subjective assessment bias. Similarly, the qualitative thematic analysis of the free-text survey responses was performed by a single coder. Because multiple coders were not used, intercoder reliability could not be established, which may have introduced subjective interpretation bias into the qualitative findings.

Although the use of PrepCheck was a strength, the hybrid scoring approach converted several continuous digital measurements into an ordinal 0-3 grading scale. This may have reduced sensitivity to subtle quantitative differences between groups. The additional direction-specific TOC analysis was included to provide greater transparency regarding the only PrepCheck parameter that directly determined the rubric score. However, this analysis was exploratory and limited to TOC. Therefore, the direction-specific TOC findings should be interpreted as supportive descriptive information rather than confirmatory evidence.

Another limitation is that the assessment focused solely on the final structural outcome of the crown preparation. The qualitative findings indicated that students perceived HVRS as useful for understanding procedural workflows, but the study did not include process-related performance measures, such as bur positioning, sequence of preparation steps, or real-time error correction. Therefore, the pedagogical value of HVRS for procedural learning may not have been fully captured.

Future studies should use randomized allocation, stratify participants by baseline manual dexterity, prior simulator exposure, and baseline self-confidence, and include larger samples to improve the reliability of comparative findings. They should also apply identical timing of dexterity assessments across groups, include process-based outcomes in addition to final preparation quality, and examine whether HVRS has differential effects among students with lower baseline manual dexterity or lower initial confidence.

### Conclusion

Within the limitations of this study, the findings suggest that 3 hours of self-directed HVRS training were not sufficient to significantly enhance overall crown preparation quality on typodont teeth or improve students’ self-reported confidence in performing typodont crown preparation. There is some preliminary indication that brief HVRS training could assist students in mastering specific geometric parameters, such as TOC, during crown preparation. Based on the present findings and prior literature, HVRS may be more appropriately investigated as an adjunctive component within a blended learning model rather than as a replacement for conventional phantom-head training. Future randomized controlled trials with stratified baseline dexterity and larger sample sizes are required to determine the optimal role of HVRS in dental education.

## Appendix

Multimedia Appendix 1 Grading scale for crown preparation assessment, including PrepCheck-based thresholds and evaluation criteria for all measured parameters.

Multimedia Appendix 2 Study questionnaire assessing self-confidence, perceptions of HVRS realism and usefulness, and free-text responses.

Multimedia Appendix 3 Exploratory direction-specific analysis of PrepCheck-derived total occlusal convergence values.

## Abbreviations

AOI: area of interest
GPT: Grooved Pegboard Test
HVRS: haptic virtual reality simulator
TOC: total occlusal convergence
VR: virtual reality

## Data Availability

The datasets used and/or analyzed during the current study are available from the corresponding author upon reasonable request.
