# Supplementary material for: Evaluating the Effectiveness of Screen-Based Haptic Virtual Reality Simulators in Preclinical Prosthodontic Crown Preparation: Mixed Methods Analysis Study
Source: JMIR Form Res. 2026 Jul 8;10:e88916. doi: 10.2196/88916 (PMC13392535; doi:10.2196/88916)
Supplement: Multimedia Appendix 1 [file formative_v10i1e88916_app1.pdf]

	3	2	1	0
**Occlusal reduction**	Optimal occlusal reduction (m. zirconia 1-1.5mm). Occlusal planes well defined and accurately reproduce occlusal contours.	Occlusal reduction sufficient. Occlusal planes slightly flat or deep. 0.8-1mm, 1.5-1.8mm	Occlusal reduction deviates from the ideal by less than 0.5 mm; occlusal planes are moderately flat or deep. Sharp angles may affect mainly all-ceramic crown (ACC)	Occlusal reduction deviates from the ideal by more than 0.5 mm on more than 30% of the occlusal surface; occlusal planes severely flat or deep. Limited space, or preparation near the pulp. Sharp angles will affect the fabrication of ACC.
**Total Occlusal Convergence (TOC)**	Optimal Total Occlusal Convergence (TOC) 10-15 degrees;	TOC 8-10 or 16-20 degrees	TOC 21-30 or less than 8 degrees.	TOC above 30 degrees.
**Axial reduction**	Axial reduction is appropriate for the tooth and restorative material. Gingival contact to the adjacent tooth is at least 1.0 mm open.	Axial reduction slightly deep or shallow. Gingival margin is at approximately 1.0 mm from the adjacent tooth	Axial reduction is moderately deep or shallow on one surface and may affect the contour or appearance of the restoration. Gingival margin is 0.5 to 1.0 mm from the adjacent tooth.	Axial reduction severely deep or shallow. Adjacent tooth remains in contact or pulp would be compromised.
**Walls and surfaces of the preparation**	Walls and surfaces are smooth and well defined	Walls and surfaces slightly rough with minor irregularities but will not affect ACC	Walls and surfaces excessively rough.	Gross roughness of all walls
**Undercut**	No undercuts	Very small undercuts that can be ignored. 0-0.05mm	Small undercuts that can be successfully blocked out. 0.05-0.10mm	Undercuts will result in areas of open margin and will interfere with insertion. >0.1mm
**Margin Design and placement**	Margin design/placement is optimal	Margin design/placement is optimal, but varies slightly in width.	Margin design/placement is acceptable, but is rough and of uneven width visibly evident. Moderate "J" margin.	Margin design/placement severely deep/shallow and very rough. Width extremely uneven throughout. Severe "J" margin.
**Finish Line**	Finish line continuous, smooth, and well defined.	Finish line continuous and smooth, but slightly uneven.	Finish line moderately rough/ irregular and slightly noncontinuous.	Finish line unacceptable (non-existent, indistinct, grossly irregular).
**Neighboring tooth injuries**	No damage to adjacent tooth.	Minor damage to adjacent tooth (can be polished without changing the contact shape or contour).	Moderate damage to adjacent tooth (can be polished but will change contact shape or contour).	Gross damage to adjacent tooth that will require restoration
