# Supplementary material for: Evaluating the Effectiveness of Screen-Based Haptic Virtual Reality Simulators in Preclinical Prosthodontic Crown Preparation: Mixed Methods Analysis Study
Source: JMIR Form Res. 2026 Jul 8;10:e88916. doi: 10.2196/88916 (PMC13392535; doi:10.2196/88916)
Supplement: Multimedia Appendix 2 [file formative_v10i1e88916_app2.pdf]

Post-Training Evaluation Survey

Evaluation after completed preparation test

Please take a moment to reflect before answering; this facilitates our interpretation of the results.

1. Did you practice with SimToCare during third and fourth semester?

☐ Yes

☐ No

2. How many hours do you estimate you practiced with SimToCare?

3. Did you practice with SimToCare during the course of the project?

☐ Yes

☐ No

4. To what extent is the sensation provided by the simulator similar to drilling in a plastic tooth?

☐ To a very small extent

☐ To a rather small extent

☐ To a rather large extent

☐ To a very large extent

5. If you have drilled on a patient, to what extent do you think the sensation provided by the simulator is similar to drilling in a real tooth?

☐ To a very small extent

☐ To a rather small extent

☐ To a rather large extent

☐ To a very large extent

☐ I have not drilled in a real tooth

6. Is there anything missing in the simulator that would provide a more realistic experience?

---

7. What aspects of the simulator did you find useful or beneficial?

---

8. What aspects of the simulator did you find less useful or less satisfactory?

---

9. To what extent do you find the simulator user-friendly (functions, settings, etc.)?

☐ To a very small extent

☐ To a rather small extent

☐ To a rather large extent

☐ To a very large extent

10. To what extent do you think the simulator is useful for self-training of manual skills for a dental student?

☐ To a very small extent

☐ To a rather small extent

☐ To a rather large extent

☐ To a very large extent

11. How likely is it that you would recommend a friend to practice on the simulator?

☐ Very unlikely

☐ Rather unlikely

☐ Rather likely

☐ Very likely

12. To what extent would you like to use the simulator to practice more manual skills?

☐ To a very small extent

☐ To a rather small extent

☐ To a rather large extent

☐ To a very large extent

13. To what extent did you have confidence in your own ability to prepare tooth 16 on typodont models?

☐ To a very small extent

☐ To a rather small extent

☐ To a rather large extent

☐ To a very large extent

14. Other comments?
