# Supplementary material for: Evaluating the Effectiveness of Screen-Based Haptic Virtual Reality Simulators in Preclinical Prosthodontic Crown Preparation: Mixed Methods Analysis Study
Source: JMIR Form Res. 2026 Jul 8;10:e88916. doi: 10.2196/88916 (PMC13392535; doi:10.2196/88916)
Supplement: Multimedia Appendix 3 [file formative_v10i1e88916_app3.docx]

| **Direction** | **SIM group, mean (SD), °** | **Control group, mean (SD), °** | **Mann–Whitney U** | **Z** | ***P* value** | **Effect size r** |
| --- | --- | --- | --- | --- | --- | --- |
| Buccopalatal | 22.22 (6.24) | 25.75 (9.79) | 179.0 | −1.48 | .139 | 0.22 |
| Mesiodistal | 22.15 (8.85) | 29.10 (9.42) | 148.5 | −2.20 | .028 | 0.33 |

**Multimedia Appendix 3. Exploratory direction-specific analysis of PrepCheck®-derived total occlusal convergence (TOC) values.**

**Note:** Comparisons were performed using the Mann–Whitney U test. *P* values are two-tailed. TOC=total occlusal convergence; B-P=buccopalatal; M-D=mesiodistal.
